# Dietary diversity and micronutrient adequacy among women of reproductive age: a cross-sectional study in Southern Thailand

**DOI:** 10.1186/s40795-022-00619-3

**Published:** 2022-11-08

**Authors:** Maneerat Puwanant, Sasivara Boonrusmee, Somchit Jaruratanasirikul, Kanjana Chimrung, Hutcha Sriplung

**Affiliations:** 1 Division of Nutrition, Department of Pediatrics, Faculty of Medicine, Prince of Songkla University, Hat Yai, Songkhla, 90110 Thailand; 2 Division of Ambulatory Pediatrics, Department of Pediatrics, Faculty of Medicine, Prince of Songkla University, Hat Yai, Songkhla, 90110 Thailand; 3 Division of Endocrinology, Department of Pediatrics, Faculty of Medicine, Prince of Songkla University, Hat Yai, Songkhla, 90110 Thailand; 4 Nutrition Unit, Faculty of Medicine, Prince of Songkla University, Hat Yai, 90110 Songkhla, Thailand; 5 Epidemiology Unit, Faculty of Medicine, Prince of Songkla University, Hat Yai, Songkhla, 90110 Thailand

**Keywords:** Dietary diversity, Mean probability of adequacy, Minimum dietary diversity for women of reproductive age, Women of reproductive age

## Abstract

**Introduction:**

Adequate nutritional intake of both macronutrients and micronutrients is essential for maintaining good health throughout life, particularly for women of reproductive age (WRA). The Minimum Dietary Diversity for WRA (MDD-W), or the sum of dietary diversity, is recommended as a simple indicator to identify at-risk WRA populations. However, there are no studies on the MDD-W among WRA in Thailand.

**Objectives:**

To determine food group diversity, MDD-W, and micronutrient intake of WRA in Southern Thailand.

**Participants:**

From December 2020 to November 2021, 120 healthy, young WRA (average age 33.2 ± 4.5 years) were enrolled.

**Methods:**

A 24-h food record was used to quantitatively and qualitatively assess the food consumed. Dietary diversity was classified into ten food groups. The macronutrients and 15 micronutrients were calculated using the software program INMUCAL, which is the standard program for calculating nutrients in Thai food. The calculated intake of each micronutrient was transformed to the probability of adequacy (PA).

**Results:**

The most common foods consumed were rice, followed by meat, eggs, fruits, and vegetables. Fewer than 40% of the participants consumed beans, dairy products, vitamin A-rich fruits and vegetables, dark green vegetables, and pulses. The average MDD-W score was 5 (range 2–8). The mean caloric intake (1,865 cal/day) was adequate for non-lactating WRA but was approximately 300 kcal/day lower than the recommendation for lactating WRA. Most WRA consumed lower amounts of micronutrients than those recommended. The mean PA (MPA) of the 15 micronutrients was 0.33 (range 0.0–0.9).

**Conclusions:**

Non-pregnant WRA in Songkhla consumed adequate macronutrients but inadequate micronutrients. A nutritional education program regarding the importance of micronutrients should be provided to the public, with special attention to WRA.

## Introduction

Adequate nutritional intake of macro- and micronutrients is essential for maintaining good health throughout life, particularly in vulnerable groups such as infants, young children, adolescents, and women of reproductive age (WRA). Macronutrient and micronutrient adequacy is crucial in WRA to prevent related diseases or conditions, adverse pregnancy outcomes, and risk of fetal problems. Dietary composition and consumption differ in each region, country, tradition, and culture, and adherence to various diets can be classified as food diversity [1]. Dietary diversity is defined as the number of different food groups consumed over a given time period, mostly within 24 h. The sum of different food groups consumed over 24 h can be used as a simple indicator of nutritional adequacy in individuals in any age group. The World Health Organization issued the ‘Global Strategy for Infant and Young Child Feeding,’ which is a guideline for the appropriate feeding of infants and young children [2]. Since 2002, the Infant and Child Feeding Index (ICFI), developed by Ruel and Menon, has commonly been used to assess child feeding practices with respect to breastfeeding and food group diversity via a summary index [3]. Various studies have shown that ICFI is associated with the nutritional status and growth of children [4–6].

In 2010, Arimond et al. developed simple food group diversity indicators that were assessed based on the number of different food groups and different food types within food groups consumed over a reference period for the assessment of micronutrient adequacy in the diet of women [7]. Promoting dietary diversity or, in less technical terms, the consumption of a wide variety of food groups has been suggested as a strategy to alleviate the inadequate intake of micronutrients. Subsequently, the ‘Minimum Dietary Diversity for Women of reproductive age’ (MDD-W), a dichotomous indicator, was developed and validated as a proxy of micronutrient adequacy [8]. MDD-W can be used as a tool for several purposes, including surveying women’s diet quality at the community level, identifying and monitoring at-risk populations, and analyzing the effect of intervention programs and national policies [8]. At present, for WRA, dietary diversity is a qualitative measure of food consumption, and MDD-W is recommended by the Food and Agriculture Organization of the United Nations (FAO) as a key nutrition-sensitive indicator [9].

Many studies in developing countries have shown that the WRA population is at high risk of specific micronutrient deficiencies, which can affect their health. Moreover, if these women become pregnant, such micronutrient deficiencies will affect pregnancy outcomes (vitamin A deficiency can result in premature birth), with effects on the fetus that can become permanent (iron deficiency results in fetal growth restriction, and folate deficiency is associated with neural tube defects) [10]. Thailand is a South-East Asian country that is in transition from a low- to a middle-income country, and it has the double burden of malnutrition and increased prevalence of obesity [11]. To date, there are no studies on the dietary diversity score and micronutrient intake among WRA in Thailand. Therefore, this study aimed to assess and analyze dietary diversity among WRA in Southern Thailand. To the best of our knowledge, this is the first study in Southern Thailand to use ten common food groups and analyze macro- and micronutrient adequacy. The primary aim of this study was to determine food group diversity, MDD-W, and micronutrient intake. The secondary aim was to clarify the association between MDD-W and the nutritional status of WRA in Southern Thailand.

## Methodology

### Sample size calculation and ethics consideration

This study was undertaken as part of a study of nutritional intake and vitamin D insufficiency in infants (age 6–12 months) and their mothers. The sample size calculation was based on the 30% prevalence of vitamin D deficiency and iron deficiency anemia in young children reported in the South-East Asian Nutrition Surveys (SEANUTS), [12, 13] with a study power of 80%. The required sample size was calculated to be 95 mothers with their infants. We expanded the recruitment to 120 participants to increase the power of the study to approximately 90%.

The protocol for this study was approved by the Institutional Review Board and Ethics Committee of Songklanagarind Hospital, Prince of Songkla University (REC 63–358-1–1). Written informed consent was obtained from the participants who were non-pregnant WRA.

### Participants

From December 2020 to November 2021, 120 healthy young mothers (no underlying diseases nor chronic illnesses, no medication use) who brought their infants aged 6–12 months for routine vaccination at the Well Child Clinic of Songklanagarind Hospital agreed to participate in this study and were scheduled for enrollment. The demographic data of the participants were collected, including the level of education, occupation, family income, current medication, and whether they were breastfeeding or not. The participants were instructed to record the details of the type and amount of food intake within the 24-h period before the scheduled date. On the scheduled date, their weight and height were measured, and they underwent a general examination. Moreover, the 24-h food intake record was rechecked by a dietitian. The body mass index (BMI) was calculated for each participant as weight in kilograms divided by height in squared meters.

### Twenty-four-hour food intake record

The 24-h food record used in this study was an open-ended list regarding various parameters for quantitatively and qualitatively assessing the food consumed over 24 h the day before the scheduled date. Each participant was instructed to record all food consumed, including the ingredient and amount (or portion) of each main food (rice/noodles/bread), meat, eggs, vegetables, milk, fruits, and desserts in addition to in-between meals (snacks, sweetened juice, tea, coffee, or carbonated drinks, etc.) and water. For outside food consumption, they were asked to describe the type, content, and amount (or portion). The participants were asked to record food intake at the time the foods were consumed or immediately after they finished the meal to minimize recall bias. Medication use (such as multivitamins and iron supplements) was also included in the food intake list. On the scheduled date, the participants underwent a detailed interview regarding their food records, and the records were rechecked by an experienced dietitian for 10–15 min to ensure the accuracy of the records, including the types and ingredients and amounts of each food consumed (estimated using common containers of different sizes e.g., bowls, plates, packages, cups, and glasses).

### Dietary diversity, food groups, and MDD-W

Dietary diversity was classified into ten food groups: 1) grains, white roots and tubers, and plantains (also known as starchy staples); 2) pulses (beans, peas, and lentils); 3) nuts and seeds; 4) dairy; 5) meat, poultry, and fish; 6) eggs; 7) dark green leafy vegetables; 8) other vitamin A-rich fruits and vegetables; 9) other vegetables; and 10) other fruits [14, 15]. Sugar-containing beverages, fats, and oils were not included as food groups because these categories of food do not contain micronutrients, but they were included in energy intake calculations. The information obtained was used to calculate the MDD-W score for each woman following the FAO and FHI 360 guidelines [9, 10]. Dietary diversity is a dichotomous variable with a value of 1 if the women consumed each of the 10 food groups or 0 otherwise. The MDD-W score is the sum of food groups consumed among the 10 required food groups, with values ranging from 1 to 10.

The food groups included in MDD-W mostly reflect diet quality with the probability of achieving minimum micronutrient adequacy across 15 important micronutrients: vitamin A, thiamine, riboflavin, niacin, vitamin B6, vitamin B12, vitamin C, vitamin E, calcium, phosphorus, magnesium, iron, copper, selenium, and zinc.

### Calculation of nutritional intake

The intake of macronutrients (carbohydrate, protein, and fat) and the 15 micronutrients was calculated based on the 24-h food record using the software program INMUCAL (Mahidol University, Thailand), which is the standard program for calculating macro- and micronutrient intake in Thai food [16].

### Selection of nutrients

We compared micronutrient adequacy using two methods: 1) with 11 selected micronutrients with public health and research relevance (vitamin A, vitamin B1 [thiamine], vitamin B2 [riboflavin], vitamin B3 [niacin], vitamin B6 [pyridoxine], vitamin B12 [cobalamine], vitamin C, vitamin E, calcium, iron, and zinc) and 2) with 15 micronutrients (the 11 micronutrients plus phosphorus, magnesium, copper, and selenium) calculated with the INMUCAL software program. In this study, iodine was not considered in the dietary analysis because of differences in iodine concentration in various household salts and fish sauces, which could have introduced calculation errors. Vitamin D was not included since the major source of vitamin D is cutaneous synthesis from direct sunlight exposure, and 97.5% of our participants were found to have very low vitamin D intake ranging from 0 to 1 μg/day. Only three participants had vitamin D intake > 5 μg/day, and the main source was vitamin D-fortified milk.

### Estimating the probability of micronutrient adequacy

To determine the adequacy of the intake of the micronutrients in our participants, it might have been preferable to use the Estimated Average Requirement (EAR), which is a recommendation for the average daily nutrient intake that is estimated to meet the requirements of 50% of the healthy individuals in a group. The RDI is higher than the EAR as it accounts for a safety factor to meet the needs of most healthy individuals in a population, which can lead to overestimating the probability of adequacy. However, the use of EAR in our study had some limitations. First, data on EARs in the Thai population is limited as it includes only 4 micronutrients (iron, zinc, vitamin A and vitamin C). Second, we have no data on the bioavailability percentage or the type of diet to compare for each micronutrient. Another limitation is that the INMUCAL software used for nutrient calculation is based on the RDI, not the EAR. Therefore, we decided to use the Thai RDI values as the reference for nutrient calculation.

The calculated total intake of each micronutrient from the 24-h food record of each participant was transformed into the probability of adequacy (PA). To calculate nutritional adequacy, we preferred the median to the mean because the mean could introduce calculation errors with regard to nutrient over- and/or under-consumption. An intake ≥ 100% of the Thai recommended daily intake (RDI) [17] was rated 0.1, and an intake < 100% was rated 0 [7, 8, 18]. The sum of all adequate micronutrients in each participant was then calculated to a maximum of 1.0. Hence, the mean PA (MPA) for micronutrient intake in an individual had a range of 0 to 1.

### Statistical analysis

Demographic data are expressed as numbers and percentages (categorical variables) and as median with interquartile ranges (continuous variables). Weight, height, and BMI are reported as mean ± standard deviation. The intake of each of the ten food groups is shown as a number and percentage. The intake of macro- and micronutrients is shown as the median and mean ± standard deviation. Student’s t-test was used to compare differences in nutritional intake between lactating and non-lactating mothers. Analysis of variance was used to compare nutritional intake between the three BMI groups; underweight, average, and obesity. Spearman’s rank coefficient was used to identify the correlation between MPA and the number of food groups consumed.

## Results

### Participants

The average participant age was 33.2 ± 4.5 years, with an average weight and BMI of 59.5 ± 11.1 kg and 23.45 ± 4.04 kg/m^2^, respectively. Sixty-nine participants (57.5%) were breastfeeding their infants. Seventy-seven percent had graduated from college or university. Most participants were from middle- to high-income families, as their average income was approximately 40,000 Baht/month (1,300 USD/month) (Table 1). None of our participants reported vitamin or other nutrient supplementation.

**Table 1:** Characteristics of the 120 participants

**Characteristic**	Mean ± S.D	Median	Range
Age, years	33.2 ± 4.5	33.2	22–40
Measurement			
Weight, kg	59.5 ± 11.1	58.0	55–90
Height, cm	159.2 ± 5.5	159.0	152–168
Body mass index, kg/m^2^	23.45 ± 4.04	22.65	15.4–38.4
Level of education, n (%)			
Secondary school	27 (22.5)	-	-
College/university	93 (77.5)	-	-
Family income, Baht/month,	39,812 ± 24,486	35,000	15,000–180,000

### Dietary diversity, food groups, and MDD-W

**Fig. 1:**
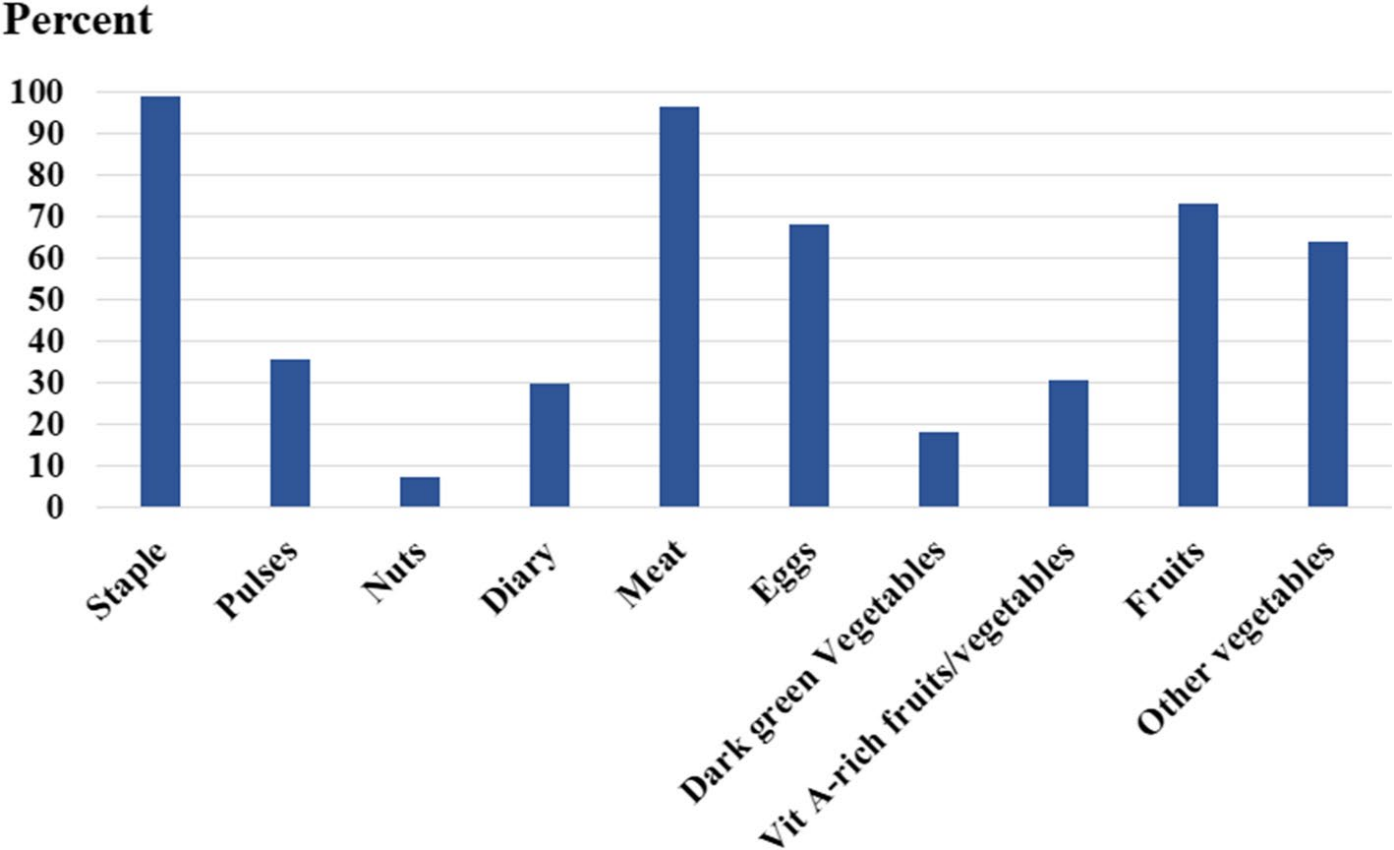
Percentage of intake of the ten food groups among women of reproductive age

The common nutritional food groups consumed by participants were staple foods, mainly rice (99.2%), meat (96.7%), eggs (68.3%), fruits (73.3%), and vegetables (64.2%). Fewer than 40% consumed beans (35.8%), dairy products (30%), and vitamin A-rich fruits and vegetables (30.8%), and very few participants consumed dark green vegetables (18.3%) and pulses (7.5%) (Fig. 1).

**Fig. 2:**
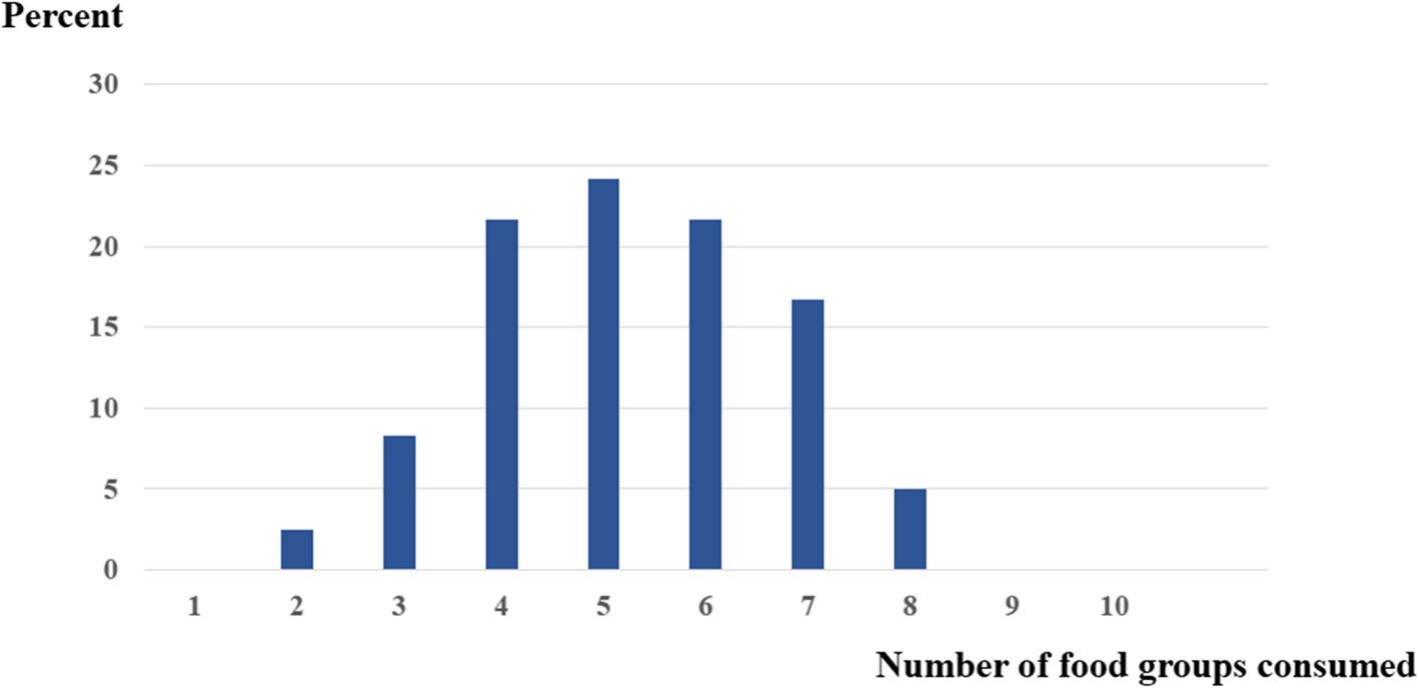
Number of food groups consumed among women of reproductive age

The average sum of food groups consumed or the MDD-W score of our participants was 5 (range 2–8) that included the 5 common aforementioned food groups (Fig. 2); 2.5%, 3.8%, and 21.7% of our participants consumed 2 (rice with meat), 3 (rice, meat, and vegetables), and 4 (rice, meat or egg, vegetables, and fruits) food groups, respectively, and 16.7% and 5% of our participants consumed 7 and 8 food groups, respectively.

### Nutrient intake calculation

The mean and median caloric intake of our participants was 1865 and 1898 cal/day, respectively (range 623–3303). The food intake of our participants notably varied in amount and type. The mean, median, and range of the nutritional intake are shown in Table 2. As shown in Table 2, the total caloric, protein, carbohydrate, and fat intakes were adequate, with appropriate rates of carbohydrate (50–55%), protein (15–20%), and fat (30–35%) intake; however, most micronutrient intakes were inadequate, including those of calcium, iron, magnesium, vitamin A, vitamin B6, vitamin B12, and vitamin E. Animal organs such as cooked liver, which are rich in minerals and vitamins, were consumed by 10% of our participants and were included in the protein group.

**Table 2:** Nutrient intake of participants, percentage of nutrient intake versus recommended daily intake (RDI) in Thailand, and the number of participants with ≥ 100% of the Thai RDI

	RDI Thailand 2020	Actual intake			Intake in % RDI	No. (%) of women with intake ≥ 100% of RDI
		Median	Mean	Range	Median	
Total calories, kcal	1,780	1,898	1,865	623–3,303	107.3	79 (65.8)
Carbohydrate, g	240	240.7	249	28–528	100.2	63 (52.5)
Protein, g	55	71.2	73	10–147	137.5	70 (58.3)
Fat, g	65	62.1	63	7–140	95.3	58 (48.3)
Saturated fat, g	20	18.2	20.2	0.1–61.7	92.3	56 (46.7)
Cholesterol, g	300	353.3	387.9	1.8–990	117.8	72 (60.0)
Calcium, mg	800	447.8	519	51–2,282	56.0	19 (15.7)
Phosphorus, mg	800	824.3	831.9	10–2,345	118.6	81 (67.5)
Iron, mg	20	10.1	10.7	1.2–29.5	41.6	4 (3.3)
Potassium, mg	2,050	1,634.5	1,796	167–4,169	-	-
Sodium, mg	2,400	3,044.0	3,249	344–8,590	-	-
Copper, mg	1.3	0.82	0.89	0.07–7.15	92.1	46 (38.3)
Magnesium, mg	250	62.6	59.6	1.6–240	24.1	3 (2.5)
Selenium, μg	55	51.6	55	6.1–98	93.2	55 (45.8)
Zinc, mg	9.7	4.9	5.2	1.1–11.6	68.6	22 (18.3)
Vitamin A, μgRE	600	384.6	627	19.4–4,687	63.8	32 (26.7)
Vitamin D, μg	5	0.5	0.92	0–8.5	10	3 (2.5)
Vitamin B1, mg	1.1	1.02	1.45	0.1–10	92.1	56 (46.7)
Vitamin B2, mg	1.1	1.27	1.46	.2–4.2	114.1	74 (61.7)
Vitamin B6, mg	1.3	0.57	0.69	0.1–3.4	42.5	9 (7.5)
Vitamin B12, μg	2.4	1.4	2	0–10	60.2	33 (27.5)
Vitamin C, mg	85	60.1	105	0–929	82.3	50 (41.7)
Niacin, mgNE	14	16.4	17.3	1.1–51.9	117.1	74 (61.7)
Vitamin E, mgTE	11	0.27	0.44	0.01–4.62	16.2	3 (2.5)
Water, mL	1750–2,625	987	1,048	390–2,650	-	-
Energy distribution from carbohydrate, %	45–65	53.8	53.3	23.1–81.7	-	-
Energy distribution from protein, %	15–20	16.2	16.2	8.6–32.9	-	-
Energy distribution from fat, %	20–35	30.5	30.5	8.5–50.8	-	-

### Estimating the probability of adequacy

**Fig. 3:**
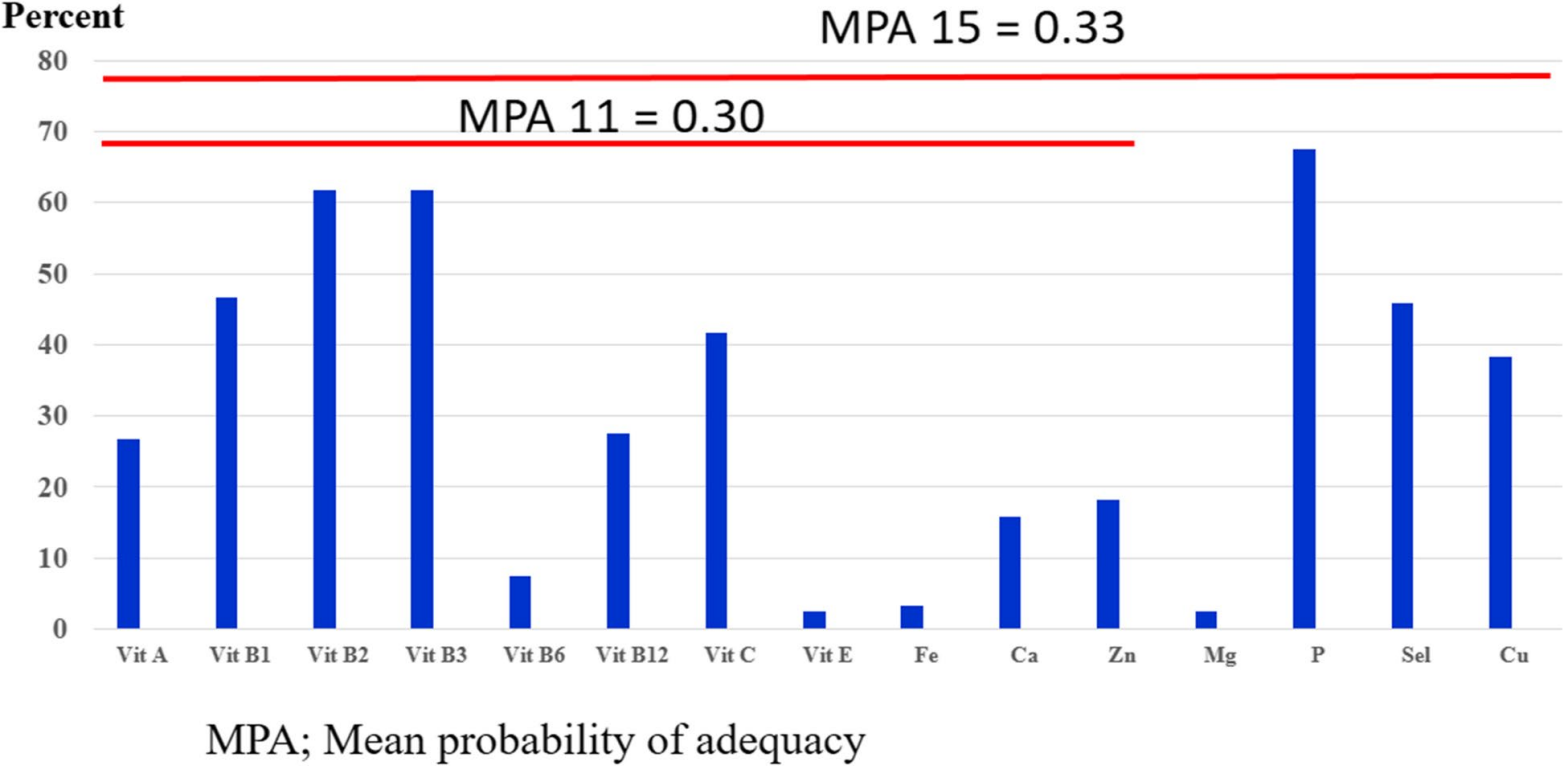
Percentage of micronutrient intake per the Thai recommended daily intake

Nutrient intake is expressed in grams (g), milligrams (mg), or micrograms (µg). Percentages of participants with ≥ 100% of the Thai RDI for each micronutrient are shown in Table 2. Of the 15 micronutrients, the percentage of adequate micronutrient intake was highest for phosphorus (67.5%), followed by vitamin B2 (61.7%), vitamin B3 (61.7%), vitamin B1 (46.7%), selenium (45.8%), and vitamin C (41.7%) (Fig. 3).

**Fig. 4:**
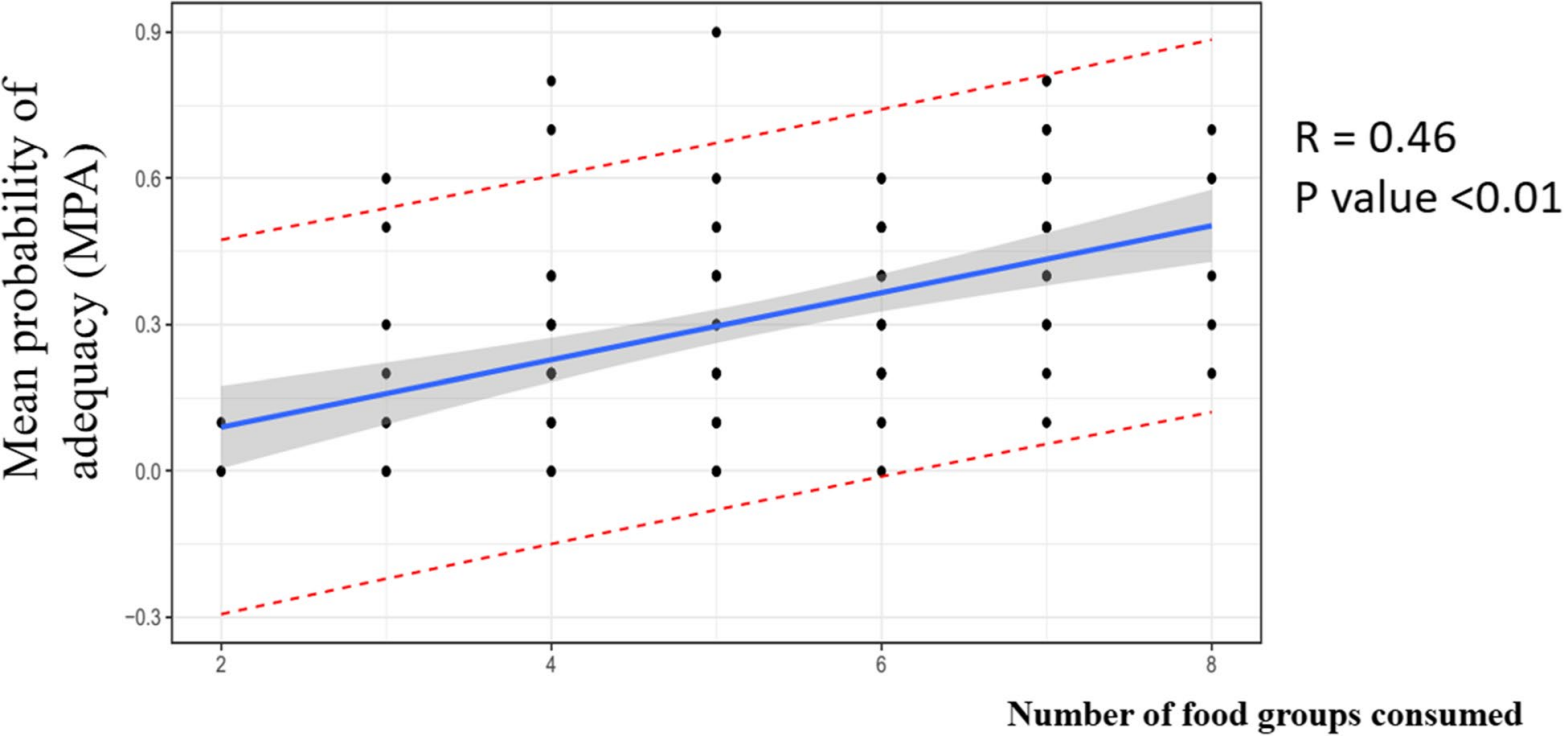
The relationship between the mean probability of adequacy (MPA) and the number of food groups consumed

After the PA of each micronutrient according to the Thai RDI was transformed to 0 and 0.1, the sum of the adequate intake of the 11 and 15 micronutrients by each participant was calculated as the MPA. We found that the average MPA for the 11 and 15 micronutrients was nearly the same at 0.3 and 0.33, respectively. MPA significantly correlated with the number of food groups consumed, as shown in Fig. 4 (*r* = 0.46, *P* < 0.001).

### Nutrient intake according to BMI and lactating status

The participants were divided to the following three groups according to their BMI [19] underweight (< 18.5 kg/m^2^; *n* = 11), normal (18.5–24.9 kg/m^2^; *n* = 70), and obese (≥ 25.0 kg/m^2^; *n* = 39). They were also divided into lactating (*n* = 69) and non-lactating (*n* = 51) groups to determine whether there were differences between these groups in nutritional intake. According to the Thai RDI recommendation, lactating women of infants aged 6–12 months need more calories and protein intake to meet their nutritional needs while breastfeeding of 300 kcal and 13 g per day, respectively [17]. Therefore, the recommended calories and protein intake for lactating women of infants aged 6–11 months in our study were 2080 kcal and 68 g per day. We found no significant differences in the total amount of caloric, macronutrient, or micronutrient intake among women according to educational level or family income. The comparisons of nutritional intake according to BMI and lactation are shown in Tables 3 and 4, respectively, for only the total caloric and macronutrient intake.

**Table 3:** Comparison of calorie and macronutrient intake with respect to body mass index (BMI)

	BMI (kg/m^2^)			*P*-value
	< 18.5 (*n* = 11)	18.5–24.9 (*n* = 70)	≥ 25.0 (*n* = 39)	
Calories, kcal	1,800	1,885	1,846	0.87
Protein, g	69.4	72.3	75.5	0.71
Carbohydrate, g	232	257	239	0.49
Fat, g	65.9	63.6	62.2	0.92

**Table 4:** Comparison of calorie and macronutrient intake between lactating and non-lactating women of reproductive age

	Lactating women (*n* = 69)		Non-lactating women (*n* = 51)		*P*-value
	RDI Thailand 2020	Median intake	RDI Thailand 2020	Median intake	
Calories, kcal	2,080	1,898	1,780	1,873	0.88
Protein, g	68	72.3	55	74.1	0.68
Carbohydrate, g	-	240	240	250	0.56
Fat, g	-	64.3	65	62.1	0.66

## Discussion

The results of this study showed that the intake of macronutrients (carbohydrate, protein, and fat) by non-pregnant WRA was adequate, with a daily energy intake of 1,800 kcal, which is approximately 100% of the Thai RDI, and an MDD-W score of 5 (rice, meat, egg, fruit, and vegetable). The distribution of the energy contribution was appropriate for carbohydrates, protein, and fat (50–55%, 15–20%, and 30–35%, respectively). However, the 1800 kcal daily energy intake was not sufficient for breastfeeding mothers, who accounted for approximately 60% of the participants. According to the Thai RDI, breastfeeding mothers should consume 600 kcal/day during the first 6 months and 300 kcal/day after 6 months of lactation; therefore, the median energy intake of these participants was approximately 300 kcal/day lower than the RDI. The prevalence of an inadequate intake of many vitamins was high in our WRA population (approximately 60% for vitamin C, 73% for vitamin A, 90% for vitamin B6, 70% for vitamin B12, and nearly 100% for vitamin E). Additionally, the intake of crucial minerals, such as calcium, iron, magnesium, zinc, and copper, was far below the RDI. The average MPA of the 11 and 15 micronutrients was only 0.30 and 0.33, respectively. Our findings regarding the inadequate intake of many micronutrients are generally consistent with previous reports concerning pregnant and non-pregnant women in Southern Thailand [20–22]. There were no significant differences in the total caloric, macronutrient, or micronutrient intake among women with regard to educational level, family income, BMI, or lactation. This finding was consistent with our previous study on pregnant women in that most participants took a similar amount of their daily regular food intake of macronutrients regardless of different levels of family income and BMI status [21]. Another explanation was that the underweight participants might over-report, whereas the obese participants might under-report their actual intake.

Micronutrient adequacy is essential for good health, particularly for WRA, in terms of preventing related diseases or conditions (such as iron deficiency, zinc deficiency, vitamin B1 deficiency), adverse pregnancy outcomes, and risk of fetal problems (iron and folate deficiency). Micronutrient deficiencies are highly prevalent worldwide in all age groups, particularly in the middle- to low-income countries, including South-East Asian countries [23, 24]. In Thailand, the prevalence of many micronutrient deficiencies in vulnerable age groups remains unknown. Previous studies in Southern Thailand focused on specific micronutrient inadequacy, e.g., iodine or iron deficiency and iron deficiency anemia, in most pregnant women [23–25].

Thailand has a wide variety of foods, fruits, and vegetables throughout the year [26], and Thai people are generally aware of the fact that consuming the five main food groups (staple, meat [all kinds of meat including egg and milk], fat, fruits, and vegetables) can provide adequate macro- and micronutrients [27, 28]. Our study showed that the MDD-W score for Thai WRA was 5, indicating the consumption of the aforementioned five main Thai food groups. The main source of staple was Thai rice enriched with vitamin B1, niacin, and selenium [17]. The main sources of meat were poultry, pork, egg, and milk. Animal organs, which are rich in minerals and vitamins [17], were included in the meat group but were consumed by only 10% of the participants. The remaining food groups (beans, dairy products, vitamin A-rich fruit and vegetables, dark green vegetables, and pulses) were only consumed by 10–40% of WRA, resulting in an inadequate micronutrient intake as shown by an MPA of only 0.33. One reason for the poorer consumption of the rest of the ten food groups is that Thai people generally believe that these remaining groups are part of the five main Thai food groups (such as milk in the meat group and vitamin A-rich fruits and vegetables, dark green vegetables, and nuts and pulses in the fruits or vegetables group). Moreover, Thai people consume nuts as a snack; thus, they are not consumed daily. Therefore, the five main Thai food groups, consisting of any kind of meat, fruits, and vegetables, are incorrectly considered to contain sufficient macronutrients and micronutrients, and consuming a low amount of dark green vegetables results in some mineral deficiencies (magnesium and iron). The low percentage of pulse and nut intake resulted in magnesium, calcium, and vitamin E deficiency, and an inadequate amount of animal organ intake resulted in iron, zinc, vitamin B2, and vitamin B12 deficiency [17]. Considering the misunderstanding surrounding the five main Thai food groups, the Department of Health, Ministry of Public Health, Thailand, launched the updated ‘Food-based dietary guidelines – Thailand’ and renamed it ‘Nutrition flag, Thailand’; here, regarding the five main Thai food groups, milk is clearly differentiated from meat, and nuts and legumes are clearly differentiated from vegetables in the fruit and vegetable group [29].

In Thailand, there have been various national programs and industrial campaigns involving a collaboration of the academic, public, and private sectors to alleviate micronutrient deficiencies. Since 1990, several national programs for controlling iodine deficiency disorders (IDD) have been implemented in Thailand [11] Since 2011, ‘Triferdine’ (iron 60 mg, folate 400 μg, and iodine 150 μg) has been distributed to every pregnant woman in antenatal care clinics to eliminate iron and iodine deficiency during pregnancy [24, 30].

In 2013, a 2-year project (SMILING; Sustainable Micronutrient Interventions to Control Deficiencies and Improve Nutritional Status and General Health in Asia), funded by the European Union (France, Denmark, UK, and the Netherlands), aimed to enhance strategies to improve micronutrient status in five South-East Asian countries (Cambodia, Indonesia, Laos, Thailand, and Vietnam) by organizing a meeting of policymakers, researchers, and stakeholders to resolve specific micronutrient deficiencies that were highly prevalent in South-East Asia and had negative health impacts (vitamin A, folic acid, vitamin B12, iron, zinc, and iodine). Micronutrient adequacy can be accomplished by consuming an appropriate combination of a wide variety of foods, including vitamin A-rich vegetables and fruits, dark green vegetables, nuts, pulses, dairy products, and eggs. Another method suggested for sustainable micronutrient intake is food fortification in staple foods. During the 2-year SMILING project, a special meeting on food fortification was organized, and they found that staple food fortification was difficult in Thailand, with the main limiting factors being the organoleptic changes in the color and taste of the fortified products and problems in selecting the best fortificants in term of stability, bioavailability, and price [31, 32]. Therefore, micronutrient adequacy in Thai WRA and in the general population still remains a challenge.

Many recent reports have demonstrated a global trend toward an increase in obesity and being overweight while undernutrition remains a problem, the so-called ‘double burden of malnutrition,’ in low- to middle-income countries in Africa and South-East Asia [33–35]. When the ‘double burden of malnutrition’ is accompanied by micronutrient deficiencies, a ‘triple burden of malnutrition’ results [36–38]. This occurs because people lack adequate knowledge of optimal nutritional intake and may purchase low-cost, energy-rich macronutrients that are low in micronutrients.

Tracking dietary diversity and quality could guide nutritional interventions that will help ensure food security. According to the FAO in 2016, MDD-W was proposed as a single indicator to assess dietary quality in WRA, and it has been widely used in low- and middle-income countries in Asia and Africa. However, to the best of our knowledge, no studies have used this tool in Thailand. According to this methodology, women with an MDD-W score > 5 are more likely to meet their micronutrient intake recommendations than their counterparts. However, our study demonstrated that WRA with an MDD-W score of 5 still had micronutrient inadequacy.

To assess dietary assessment diversity and nutrient intake, we preferred to use a 24-h food record as it minimizes the error from recall bias. There are various methods of dietary assessment used in research studies, including 24-h food recall, 24-h food record, dietary history (7-day diary, 3-day food record/recall), and food frequency questionnaires. Each method is based on self-reporting, which has its inherent strengths and limitations. The 24-h food recall and food frequency questionnaires are practical and easy to use, but their main limitation is the recall bias due to the underreporting of packaged foods, drinks, or reporting the wrong size of food container [39, 40]. Several days of food record or recall can provide more details on daily nutrient intake data; however, this dietary assessment method is time-consuming and may induce the burden on the participant to collect information. Moreover, an in-depth interview of multiple days of record/recall required approximately 60–90 min by a skilled dietitian for each participant. To minimize the recall bias and burden of multiple days of food record/recall, we decided to use a 24-h food record to collect the details of all kinds of food consumed in 24 h.

The main strength of the study is that the calculation of macro- and micronutrient intake was based on the Thai software program INMUCAL, which is the standard program for the accurate calculation of macronutrients and micronutrients in specific Thai food [17]. The main limitation of this study was that it was a hospital-based research at the time of the COVID-19 pandemic (year 2021) but not during the lockdown period; thus, all participants in our study belonged to urban areas and were mostly educated with relatively high family incomes. Despite this limitation, we demonstrated multiple inadequate micronutrient intakes, and these findings could reflect the high percentage of multiple inadequate micronutrient intakes in the general population. Another limitation is that the 24-h food record is subject to bias because it relies on participants reporting their own intake, and they might under- or over-report their actual intake. To the best of our knowledge, there is no gold standard method for preventing the underreporting or over-reporting of nutritional intake or detecting variations in day-to-day nutritional intake [40]. Therefore, we aimed to minimize this bias via an in-depth interview of participants by an experienced dietitian to assess the 24-h food record to obtain accurate data regarding the ingredients and portion/size of food consumed.

In summary, this study found that non-pregnant, non-lactating WRA in the Songkhla province consumed adequate amounts of macronutrients while the lactating WRA consumed approximately 300 kcal/day of macronutrients less than the Thai RDI. Most WRA consumed lower amounts of micronutrients than those recommended. These micronutrient deficiencies should be regarded as public health risks. A nutritional education program regarding the importance of micronutrients and information on appropriate foods and adequate diets should be provided to the public, with special attention to WRA. Further research concerning adequate MDD-W and MPA should be undertaken after a nationwide nutritional education program is successfully implemented.

## Data Availability

The data are not publicly available due to it was funded by the Southern Thailand Institute of Research and Development and some data contained information that could compromise the privacy of research participants However, The data used to support the findings of this study are available from the corresponding author (SJ) upon request.
